# Urban scaling and the regional divide

**DOI:** 10.1126/sciadv.aav0042

**Published:** 2019-01-30

**Authors:** Marc Keuschnigg, Selcan Mutgan, Peter Hedström

**Affiliations:** Institute for Analytical Sociology, Linköping University, Norra Grytsgatan 10, 601 74 Norrköping, Sweden.

## Abstract

Superlinear growth in cities has been explained as an emergent consequence of increased social interactions in dense urban environments. Using geocoded microdata from Swedish population registers, we remove population composition effects from the scaling relation of wage income to test how much of the previously reported superlinear scaling is truly attributable to increased social interconnectivity in cities. The Swedish data confirm the previously reported scaling relations on the aggregate level, but they provide better information on the micromechanisms responsible for them. We find that the standard interpretation of urban scaling is incomplete as social interactions only explain about half of the scaling parameter of wage income and that scaling relations substantively reflect differences in cities’ sociodemographic composition. Those differences are generated by selective migration of highly productive individuals into larger cities. Big cities grow through their attraction of talent from their hinterlands and the already-privileged benefit disproportionally from urban agglomeration.

## Teaser

Population characteristics and selective migration explain around half of the previously reported superlinear urban scaling.

## INTRODUCTION

**Fig. 1:**
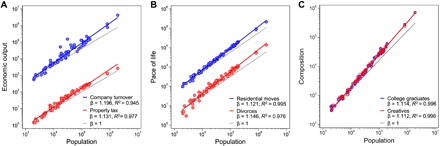
Scaling relations of urban indicators across Sweden’s 75 labor market areas in 2012. (**A**) In line with previous research (*1*–*3*), we find superlinear growth in indicators of economic output such as total company turnover [blue: β = 1.196 ± 0.048 (95% confidence interval), *R*^2^ = 0.945] and the property tax collected in each labor market area (red: β = 1.131 ± 0.050, *R*^2^ = 0.977), both measured in millions of Swedish krona. Gray lines indicate proportional relations (β = 1); the colored lines show estimates of β from a linearized model (see Eq. 1 in Materials and Methods). (**B**) Acceleration of the pace of life is apparent from the number of residential moves (blue: β = 1.121 ± 0.022, *R*^2^ = 0.995) and the number of divorces (red: β = 1.146 ± 0.051, *R*^2^ = 0.976). (**C**) Cities also differ in their composition: The number of college graduates (blue: β = 1.114 ± 0.019, *R*^2^ = 0.996) and of employees in creative jobs (red: β = 1.112 ± 0.017, *R*^2^ = 0.996) likewise follow scaling relations.

An influential research tradition quantifies urban agglomeration effects of wealth and social change as scaling relationships (*1*–*7*): Attributes of cities change with their size, and a power-law function *Y*(*N*) ~ *Y*_0_*N*^β^ captures these associations, where *Y* represents a socioeconomic quantity’s city-wide total and *Y*_0_ and β are constants to population size *N*. The parameter β is a scale-invariant elasticity, indicating the percentage change in *Y* following a 1% increase in *N*. Doubling city size, for example, reportedly raises total income by roughly 115%—or 15% per capita—suggesting that urbanites become wealthier as their cities grow. Corroborating previous research (*1*–*3*), we find similar superlinear scaling relations for economic outputs and measures of the pace of life in Swedish cities (Fig. 1).

Existing models explain superlinear scaling parameters with reference to increases in social interconnectivity with rising urban density (*2*–*5*, *8*–*10*). These explanations view superlinear growth as an endogenous process and thus as an emergent property of city life. This interpretation of urban scaling resonates well with sociological descriptions of cities as social accelerators easing the flow of information, behaviors, and ideas (*11*–*13*) and is in line with the notion of density externalities in the research on agglomeration economies (*14*–*16*). However, these literatures have also highlighted how the composition of local populations and their workforce skills vary with city size (*17*–*20*), how complementaries among occupations and business types affect urban outputs (*21*–*23*), and how cities attract talent, tipping urban populations toward higher productivity (*24*–*26*). These findings raise the question of what role cities’ population compositions play for the observed superlinear urban scaling.

Using Swedish register data with unique granularity, we explore how much of β can truly be attributed to increased social interconnectivity in cities. The Swedish data confirm the previously reported scaling relations on the aggregate level (Fig. 1), but they provide better information on the micromechanisms responsible for observed urban scaling. Our geocoded microdata capture the dissimilarities in sociodemographic composition between labor market areas of different size. Focusing on the scaling relation of wage income, we find that social interactions at best explain 61% of the scaling parameter, and differences in population characteristics between metropolitan areas crucially add to superlinear urban scaling. Fueled by selective migration from smaller to larger cities, these composition differences explain at least 39% of the observed scaling parameter. This finding provides a more nuanced understanding of the mechanisms underlying superlinear urban scaling. We find that big cities grow through their attraction of talent from their hinterlands and—going beyond the analysis of average scaling—that various sociodemographic groups benefit differently from superlinear growth. These findings are of considerable policy relevance and suggest that the already-privileged benefit disproportionally from urban agglomeration.

### Population descriptives

**Fig. 2:**
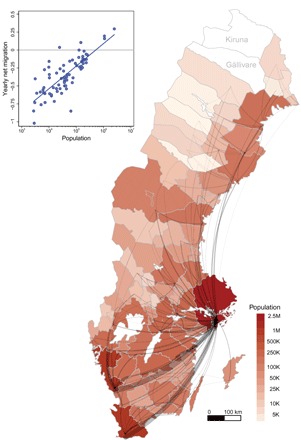
Sweden’s 75 labor market areas. Labor market boundaries reflect commuting patterns. We colored each labor market area according to its population size (2673 to 2.51 million inhabitants). The gray ties indicate migration flows from smaller to relatively larger labor markets, weighted by the numbers of movers in 2012. In absolute terms, most movers to denser urban environments appear within the country’s largest labor market areas, reflecting their overall population size. The inset plots yearly net-migration flows (inward movers–outbound movers) during 1990–2012 as a percentage of the local working-age population against the size of labor market areas [the blue line indicates a linear best fit (slope 0.136 ± 0.023, *R*^2^ = 0.623)]. We exclude Gällivare (18,307 inhabitants) and Kiruna (22,968), the mining areas in the far north, from our individual-level analyses, as their economies depend almost exclusively on the extraction of natural resources.

Statistics Sweden, the country’s central statistical office, assembled longitudinal microdata for us on Sweden’s entire population by merging administrative population registers—something possible only in countries with extensive and standardized population records. The population registers provide a detailed picture of composition differences between smaller and larger places. We use Sweden’s 75 labor market areas (Fig. 2) as a functional demarcation of metropolitan areas (*27*). In 2012, around half of the labor force lived in one of the four biggest urban areas [Stockholm (2.51 million inhabitants), Malmö (1.09 million inhabitants), Gothenburg (1.08 million inhabitants), and Linköping (0.26 million inhabitants)]. On average, the individuals in these cities are younger [−0.81 years (±0.011, 95% confidence interval)], better educated [+0.55 (±0.002) years of education], and smarter [+0.53 (±0.003) SDs in a *z*-standardized test of cognitive ability among male conscripts; mean, 0; SD, 1 (*28*–*30*)] than those in the rest of the country. Composition attributes such as the numbers of college graduates and of creative professionals themselves follow scaling relations (Fig. 1C).

There is also strong evidence for selective migration (*18*, *25*, *31*): Compared to those left behind, the educated and the smart are more likely to leave smaller places for larger labor markets. On average, those who left during 1990–2012 have 1.78 (±0.004) more years of education, and their cognitive ability is 0.42 (±0.003) SDs higher than those who stayed. Figure 2 signifies migration flows from smaller to relatively larger labor market areas, weighted by the number of movers in 2012. The Stockholm area (in the east of the country) receives the largest number of internal migrants, followed by Gothenburg’s and Malmö’s labor markets (both in the southwest). This suggests that scaling relations may reflect composition differences originating in the mobility of highly productive individuals into bigger cities. The inset, lastly, plots yearly net-migration flows during 1990–2012 as a percentage of the local working-age population against the size of labor market areas: Whereas the largest labor markets receive a net inflow of migrants, smaller places with less than 100,000 inhabitants are in constant decline (Fig. 2, inset). These changes have cumulative effects on local populations in sending and receiving regions.

## MATERIALS AND METHODS

**Fig. 3:**
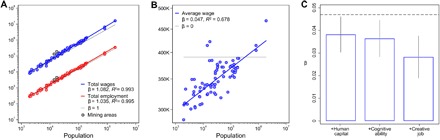
Composition effects on the scaling of wages. (**A**) Total wages of Swedish males, measured in millions of Swedish krona, scale superlinearly across labor market areas (blue: β = 1.082 ± 0.022, *R*^2^ = 0.993). So does labor market participation, measured as the total number of employees (red: β = 1.035 ± 0.019, *R*^2^ = 0.995). We exclude the mining areas Gällivare and Kiruna [gray dots (see the Supplementary Materials for robustness analyses)]. (**B**) Per-capita wage (blue) also relates above proportionally to labor market size (β = 0.047 ± 0.008, *R*^2^ = 0.678), carrying the remainder of the total scaling relation (1.035 + 0.047 = 1.082). The gray line indicates a proportional per-capita relation [β = 0 (see Eq. 2)]. (**C**) Statistically controlling for human capital, cognitive ability, and creative job characteristics further reduces the per-capita scaling relation to β = 0.028 ± 0.009 (see Eq. 3). The vertical lines indicate 95% confidence intervals, and the dashed line stands for the per-capita scaling parameter β = 0.047 without composition controls.

We estimated the scaling exponent β for city-wide totals (Figs. 1 and 3A) following standard practice (*3*): We reformulated the power-law function $Y_{j}(N)\sim Y_{0}N_{j}^{\beta }$—where *Y* is an aggregate attribute of city *j* = 1, 2,..., *M*, *N* is its population size, and *Y*_0_ is the intercept—as a linearized model

$$\text{log}\;Y_{j}=\;\text{log}\;Y_{0}+\beta \;\text{log}\;N_{j}+\epsilon_{j}$$ (1)

in which *j* runs over labor market areas and ϵ_*j*_ is a normally distributed error with zero mean. We approximated β using linear ordinary least squares regression, minimizing $\sum_{j=1}^{M}{\left(\text{log}\;Y_{0}N_{j}^{\beta }-\;\text{log}\;Y_{j}\right)}^{2}$—the sum of labor markets’ squared distances to a linear best-fit function that relates city sizes to urban outputs. The linear function’s slope equals β, and superlinear scaling implies β > 1.

For a decomposition of the total scaling relation (Fig. 3B), we then substituted a city’s average wage for its sum of wages

$$\text{log}\frac{Y_{j}}{N_{j}}=\;\text{log}\;Y_{0}+\beta \;\text{log}\;N_{j}+\epsilon_{j}$$ (2)

Changing to an “intensive” (*32*) per-capita quantity implies proportional scaling at β = 0.

**Fig. 4:**
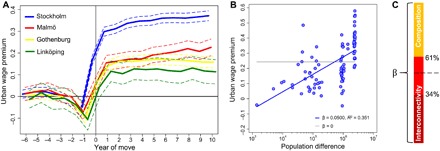
Urban wage premium approximates the upper bound of the interconnectivity effect. (**A**) Urban wage premium for movers from smaller labor market areas to the four largest in 1993–2012. The horizontal line represents movers’ counterfactual wages (at year *t* counted from the year of move) had they remained (see Eq. 4). Both the immediate (*t* = 1) and the long-term urban wage premium (*t* = 10) relate positively to population size and are largest for those entering the Stockholm labor market (+29.8% ± 2.1% at *t* = 1 and +37.2% ± 2.3% at *t* = 10); dashed lines indicate 95% confidence intervals. (**B**) There exist (72 × 73)/2 = 2628 potential combinations of origin and target labor markets in moving from a smaller to a relatively larger area. Relating the mean urban wage premium for the 100 labor market pairs with at least 200 movers to the logarithm of their difference in population size reveals a scaling relation of β = 0.050 ± 0.014, *R*^2^ = 0.351. (**C**) Our two complementary analyses reduce 34 to 61% of wages’ scaling parameter to interconnectivity effects (red). Most likely, interconnectivity explains about half of the scaling relation.

**Fig. 5:**
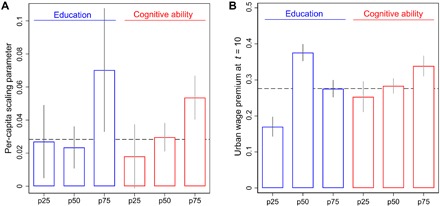
The social gradient of urban scaling. (**A**) The highly educated (per-capita scaling parameter β = 0.070 ± 0.037) and those with high cognitive ability (β = 0.054 ± 0.013) benefit most from living in urban environments. We split the study population into three groups consisting of those with relatively little (<25th percentile), intermediate (25th to 75th percentile), or high (>75th percentile) education or ability, respectively. The vertical lines indicate 95% confidence intervals and the dashed line represents the net-agglomeration effect β = 0.028 ± 0.009 from Fig. 3C. (**B**) Long-term urban wage premium is smallest for the least-educated (+17.0% ± 2.7%) and the least-able (+25.3% ± 4.3%), who thus benefit least from moving into urban environments. The dashed line is the unconditional long-term urban wage premium averaged over the trajectories shown in Fig. 4A.

Our main analyses (Figs. 3 to 5) focus on individuals’ wage earnings as a local source of income. We refrained from using personal income—typically defined as wage income plus governmental transfers—because the latter is a “mixed quantity” including transfers redistributing income from rich (large) to poor (small) areas (*33*) and may thus wash out scaling relations. To prevent bias from, for example, differences in female labor force participation, we restrict our data to fully employed Swedish-born males. We also dropped all residents from the mining areas Gällivare and Kiruna, whose wages depend primarily on the presence of natural resources. This leaves us with 1.29 million individuals nested in 73 labor market areas.

In a cross-sectional analysis—our test strategy to approximate the lower bound of the interconnectivity effect—we partialed out composition effects on the per-capita scaling of wages, first, by considering the human capital earnings function (*34*, *35*), which models individual log(wage) as the sum of years of education and a quadratic function of years of work experience. Both education (12.5 years on average; SD, 2.3) and work experience (17.1 years on average; SD, 6.0) are directly observable in the register data. Second, our study improves on otherwise seminal net-agglomeration studies from regional economics (*24*, *25*, *36*) in including a standardized measure of cognitive ability (mean, 0; SD, 1), and thus crucially extends the vector of observed individual characteristics. Third, we included a binary variable measuring each employee’s innovativeness (*18*, *37*, *38*), assigning the value 1 to each employee in a creative occupational category (0 for employees in all other occupations). In our restricted data, 47.1% work in creative jobs. To arrive at a net-agglomeration effect for Swedish wages (Fig. 3C), we estimated microlevel log(wage) regressions and included our proxies for individual productivity. Taking into account the hierarchical data structure—the 1.29 million individuals *i* are nested in 73 labor market areas *j* with different industry structures, occupational opportunities, and historical inertia—we regressed the logarithm of individual wage *y* on city size and our productivity controls in two-level random-effects regressions

$$\text{log}\;y_{ij}=\;\text{log}\;y_{0}+\beta \;\text{log}\;N_{j}+X_{ij}\gamma +\nu_{j}+\epsilon_{ij}$$ (3)

The random effect ν_*j*_ captures regional idiosyncrasies by shifting each labor market’s intercept, and ϵ_*ij*_ remains the pure residual. **X** represents the vector of composition controls covering years of education, years of experience, years of experience^2^, cognitive ability, and creative job characteristics.

Our second test strategy approximates the upper bound of the interconnectivity effect. We contrasted movers’ wages before and after they have migrated into a larger labor market area to quantify how a densely populated environment affects their productivity. Our estimation of the urban wage premium (Fig. 4A) thus rests on the wage trajectories of individuals who—between 1993 and 2012—left their native areas to work in relatively larger labor markets. Using longitudinal regressions, we modeled separate migration effects on log(wage) for each year following *i*’s move vis-à-vis a counterfactual wage had *i* stayed in his native labor market area (*39*, *40*). To identify movers’ annual wage changes relative to expected wages in their native labor market areas, we estimated

$$\text{log}\;y_{it}=M_{it}\gamma_{t}+X_{it}\delta +\alpha_{i}+\epsilon_{it}$$ (4)

The individual-specific fixed effect α_*i*_ absorbs time-constant personal characteristics such as ability and motivation, and ϵ_*it*_ represents the pure residual. **M**_*it*_ is a vector of binary variables indicating a move in a previous, current, or future year as defined by a process-time axis centered at the year of migration. In total, we included 17 binary variables: one for each of the (maximum) 6 years preceding migration (*t* < 0), one for the year of migration (*t* = 0), and one for each of the (maximum) 10 years following migration (*t* > 0). Each binary variable contrasts *i*’s wage at *t* to his wage before migration. The premigration dummies capture movers’ wage trends before they left for larger labor market areas. For each year following the move, the parameter vector γ_*t*_ indicates the returns from migration—our percentage estimate of the urban wage premium. To adjust the counterfactual for overall wage trends, the model includes not only movers (the “treated”) but also each area’s stayers (the “untreated”), who carry the value 0 in all yearly dummies throughout process time. Hence, we also used the wage data of stayers to infer how movers’ earnings would have developed had they remained in their native labor market. We further adjusted the counterfactual for changes in regional gross domestic product in native labor markets (gross domestic product in millions of Swedish krona at current prices). In addition, the vector **X** controls for yearly changes in individuals’ education, experience, experience^2^, and employment status.

## RESULTS

Our main analyses focus on wage earnings as a local source of income. We restrict our full population data to fully employed Swedish-born males and their labor-market productivity-related characteristics. We use two test strategies to approximate the lower and the upper bounds of the interconnectivity effect on the scaling parameter of urban wages. Both test strategies complement each other and only in combination provide a valid estimate of the interconnectivity effect.

### Lower-bound estimate of the interconnectivity effect

Partialing out composition effects from the wage-city size relation permits a residual approximation of the interconnectivity effect underlying urban scaling. Following this test strategy, we control for observable factors affecting the determination of individual wages, and we interpret the remaining city-size effect on wages as the consequence of increased social interactions in dense urban environments. If the aggregate scaling relation exclusively was the result of increased social interactions, then partialing out population characteristics would not affect β.

Figure 3A shows superlinear scaling not only for total wages (β = 1.082 ± 0.022) but also for the total number of employees (β = 1.035 ± 0.019). Higher rates of employment may be endogenous to city life and thus consistent with the interconnectivity explanation. On the other hand, it may reflect characteristics of those moving into bigger cities to participate in their buzzing labor markets: Considering the full working-age population of Sweden, employment is higher among those who have moved into the four biggest labor market areas (76.9% 10 years after migration) than among those areas’ native inhabitants (67.7%). Thus, bigger cities’ higher labor force participation may be an exogenous driver of the total wage scaling relation. Labor market areas’ per-capita wage (Fig. 3B) then carries the remaining part of the scaling relation (β = 0.047 ± 0.008; see Eq. 2).

We then estimate the wage-city size relation controlling for productivity-related measures of sociodemographic composition in individual-level log(wage) regressions (see Eq. 3). Including education, work experience, cognitive ability, and creative job characteristics as controls further reduces the elasticity between wages and city size: A doubling of city size results in an expected wage increase of 2.8% (±0.9%) per capita (Fig. 3C; see also table S3). This net-agglomeration effect (*24*, *25*, *36*) approximates 34% of the total per-capita scaling relation (0.028/0.082 = 0.341) and is not explainable by differences in individual characteristics and is therefore consistent with the interconnectivity explanation. Although adding more controls could further reduce the wage size elasticity, we would risk overcontrolling urban composition’s indirect consequences. These indirect consequences arise from interplay between different population characteristics, most notably increasing returns to the mixing of knowledge workers (*19*, *41*) and functional complementaries among occupations (*23*, *42*) and business types (*21*, *43*). Indirect consequences like these should not be partialed out of the net-agglomeration effect because they are rooted in social interactions and are thus consistent with the interconnectivity explanation. Hence, the residual approach provides a lower-bound estimate for the interconnectivity effect on urban scaling relations.

### Upper-bound estimate of the interconnectivity effect

For an upper-bound estimate, we quantify how a densely populated environment affects the wage trajectories of individuals migrating into bigger cities (1993–2012). We then connect the wage increases movers enjoy from urban exposure to the log(difference in population size) between their native and their target labor market area. The interconnectivity explanation predicts β to approximate the total per-capita scaling parameter for wages from the previous analysis (β = 0.082 ± 0.022). We expect 0.028 < β < 0.082 because we allow interplay between population characteristics to affect the scaling relation but, in a longitudinal analysis following individual wage trajectories over time, exclude all direct composition effects. Figure 4A illustrates our estimation of the urban wage premium for movers to the four largest labor market areas against counterfactual wages (gray line) had they remained in their native labor markets (see Eq. 4). Both the immediate (at *t* = 1) and the long-term urban wage premium (at *t* = 10) relate positively to the target area’s population size and are most pronounced for those joining Stockholm’s labor market. Moving to bigger cities thus raises wages considerably, implying that cities provide better environments for their skills including access to jobs not available in smaller places.

To approximate the interconnectivity effect on individual wages, we are interested in the urban wage premium conditional on population differences between native and target labor market areas. We focus on the long-term urban wage premium, which includes postmigration earning paths, capturing not only immediate wage benefits of big-city employment but also the accumulation of learning effects in high-density urban environments over time (*24*, *44*–*46*). There exist (72 × 73)/2 = 2628 unique ways of moving from a smaller to a relatively larger labor market. We estimate a separate mean long-term urban wage premium for each combination of potential origin and target labor markets. In Fig. 4B, we relate the mean of movers’ urban wage premiums to the logarithm of the population difference for each combination. To achieve maximally reliable estimates, we restrict the scaling analysis to the 100 labor-market combinations with ≥200 movers (representing 72,866 movers in total). We find a scaling relation of β = 0.050 ± 0.014. In this specification, 61% of the per-capita scaling parameter (0.050/0.082 = 0.611) is consistent with the interconnectivity explanation (see the Supplementary Materials for robustness analyses). It is important to note that our estimation of the urban wage premium uses data on movers that are not representative of the population as a whole. Because those who benefit most from city life are also most likely to migrate into bigger cities, we overestimate the true urban wage premium, providing an upper-bound estimate of the interconnectivity effect.

## DISCUSSION

Combining the results from our two analyses, we find that population characteristics explain between 39 and 66% of the scaling parameter for wages (Fig. 4C). The fraction of the total scaling coefficient that can be explained by interconnectivity thus ranges between 34% (based on the cross-sectional analysis) and 61% (based on the longitudinal analysis). Interpreting the mean of the interval as the most likely approximation, our results suggest that increases in social interconnectivity account for around half of the urban scaling relation. Differences in local population composition—fueled by migration from smaller to larger cities—account for the other half.

Although an early analysis of patenting activity in U.S. Metropolitan Statistical Areas suggested that composition differences may be important for observed scaling relations (*47*), this finding has been largely ignored in later publications. Our results underscore the importance of heterogeneous population characteristics in bringing about superlinear urban scaling, and we highlight a mechanism that complements the network-based explanation that currently dominates the literature. The analyses we present hence not only add to our descriptive understanding of superlinear urban scaling but correct the current and widely accepted explanation. This finding also demonstrates that the existence of an aggregate scaling relationship itself says little about the causal processes that brought it about (*48*).

Our composition-based explanation is of considerable policy relevance. On the individual level, agglomeration benefits correlate with sociodemographic background, and the already-privileged—who appear most able in absorbing density externalities—benefit disproportionally from urban agglomeration. The highly educated (per-capita scaling parameter β = 0.070 ± 0.037) and those with high cognitive ability (β = 0.054 ± 0.013) benefit most from living in urban environments (Fig. 5A). Similarly, the long-term urban wage premium is smallest for the least-educated (+17.0% ± 2.7%) and the least-able (+25.3% ± 4.3%), who thus benefit least from moving into urban environments (Fig. 5B).

On the system level, the higher than expected productivity of larger cities is only partially endogenous but depends significantly on influxes of outside talent. In our data, those moving into larger cities differ strongly from those left behind. On average, movers to larger places also exceed the native population in their target areas by +0.74 (±0.016) years of education and +0.17 (±0.011) SDs in cognitive ability, crucially contributing productivity to urban labor forces. The most-productive are more likely to leave smaller places and tend to self-select into the biggest labor market areas (see table S1B), thus magnifying broad population differences between regions. Migration flows further signify that the largest labor markets receive net inflows of migrants, whereas population sizes in smaller places decline (Fig. 2).

While interconnectivity plays an important role in bringing about superlinear urban scaling, superlinearity reflects, to a considerable extent, mechanisms previously neglected in the scaling literature. Big cities grow through their attraction of highly productive individuals from their hinterlands, and this mechanism is consequential for societies because selective migration has cumulative effects on local populations in both sending and receiving regions. Our findings are thus consistent with the increasingly uneven economic geography observed in many countries in which cities’ attraction of talent adds to growing levels of inequality between urban and rural areas.

## Supplementary Material

http://advances.sciencemag.org/cgi/content/full/5/1/eaav0042/DC1

## SUPPLEMENTARY MATERIALS

Supplementary material for this article is available at http://advances.sciencemag.org/cgi/content/full/5/1/eaav0042/DC1 Section S1. Full population data, metropolitan areas, and regional composition Section S2. Outlier analysis of urban scaling parameters Section S3. Replication of the scaling relation’s decomposition with U.S. data Section S4. Wage data and measures of individual productivity Section S5. Full tabulation of cross-sectional results Section S6. Full tabulation of the urban wage premium Fig. S1. Scaling relations of urban indicators excluding the three largest labor market areas. Fig. S2. Decomposition of the total scaling relation for wages across U.S. Metropolitan Statistical Areas. Fig. S3. Complementary analyses of the urban wage premium. Table S1. Description of Sweden’s full working-age population. Table S2. Creative jobs and the corresponding occupational codes. Table S3. Composition effects on the scaling of wage income. Table S4. Urban wage premium following a move from one of Sweden’s smaller labor market areas to one of the four largest.

## References

[1] Bettencourt L. M. A., Lobo J., Helbing D., Kühnert C., West G. B., Growth, innovation, scaling, and the pace of life in cities. Proc. Natl. Acad. Sci. U.S.A. 104, 7301–7306 (2007). 17438298 10.1073/pnas.0610172104 PMC1852329

[2] Bettencourt L. M. A., The origins of scaling in cities. Science 340, 1438–1441 (2013). 23788793 10.1126/science.1235823

[3] Bettencourt L. M. A., Lobo J., Urban scaling in Europe. J. R. Soc. Interface 13, 20160005 (2016). 26984190 10.1098/rsif.2016.0005 PMC4843676

[4] Batty M., The size, scale, and shape of cities. Science 319, 769–771 (2008). 18258906 10.1126/science.1151419

[5] G. B. West, *Scale: The Universal Laws of Growth, Innovation, Sustainability, and the Pace of Life in Organisms, Cities, Economies, and Companies* (Penguin, 2017).

[6] Schläpfer M., Bettencourt L. M. A., Grauwin S., Raschke M., Claxton R., Smoreda Z., West G. B., Ratti C., The scaling of human interactions with city size. J. R. Soc. Interface 11, 20130789 (2014). 24990287 10.1098/rsif.2013.0789 PMC4233681

[7] Ortman S. G., Cabaniss A. H. F., Sturm J. O., Bettencourt L. M. A., Settlement scaling and increasing returns in an ancient society. Sci. Adv. 1, e1400066 (2015). 26601129 10.1126/sciadv.1400066 PMC4644079

[8] Arbesman S., Kleinberg J. M., Strogatz S. H., Superlinear scaling for innovation in cities. Phys. Rev. E 79, 016115 (2009). 10.1103/PhysRevE.79.016115 19257115

[9] Pan W., Ghoshal G., Krumme C., Cebrian M., Pentland A., Urban characteristics attributable to density-driven tie formation. Nat. Commun. 4, 1961 (2013). 23736887 10.1038/ncomms2961

[10] Yakubo K., Saijo Y., Korošak D., Superlinear and sublinear urban scaling in geographical networks modeling cities. Phys. Rev. E 90, 022803 (2014). 10.1103/PhysRevE.90.022803 25215777

[11] M. Weber, *Economy and Society* (University of California Press, 1978).

[12] Wirth L., Urbanism as a way of life. Am. J. Sociol. 44, 1–24 (1938).

[13] J. Jacobs, *The Economy of Cities* (Vintage, 1969).

[14] A. Marshall, *Principles of Economics* (MacMillan, 1890).

[15] G. Duranton, D. Puga, in *Handbook of Regional and Urban Economics, Vol. 4*, V. Henderson, J.-F. Thisse, Eds. (North-Holland, 2004), pp. 2063–2117.

[16] M. Fujita, J. F. Thisse, *Economics of Agglomeration: Cities, Industrial Location, and Globalization* (Cambridge Univ. Press, 2013).

[17] B. Bishop, R. G. Cushing, *The Big Sort: Why the Clustering of Like-minded America Is Tearing Us Apart* (Houghton Mifflin, 2008).

[18] R. Florida, *The Rise of the Creative Class* (Basic Books, ed. 2, 2012).

[19] Abel J. R., Dey I., Gabe T. M., Productivity and the density of human capital. J. Reg. Sci. 52, 562–586 (2012).

[20] Alabdulkareem A., Frank M. R., Sun L., AlShebli B., Hidalgo C., Rahwan I., Unpacking the polarization of workplace skills. Sci. Adv. 4, eaao6030 (2018). 30035214 10.1126/sciadv.aao6030 PMC6051733

[21] W. Christaller, *Central Places in Southern Germany* (Prentice Hall, 1966).

[22] Quigley J. M., Urban diversity and economic growth. J. Econ. Perspect. 12, 127–138 (1998).

[23] Gomez-Lievano A., Patterson-Lomba O., Hausmann R., Explaining the prevalence, scaling and variance of urban phenomena. Nat. Hum. Behav. 1, 0012 (2016).

[24] De La Roca J., Puga D., Learning by workers in big cities. Rev. Econ. Stud. 84, 106–142 (2017).

[25] Combes P.-P., Duranton G., Gobillon L., Spatial wage disparities: Sorting matters!. J. Urban Econ. 63, 723–742 (2008).

[26] Lobo J., Mellander C., Stolarick K., Strumsky D., The inventive, the educated and the creative: How do they affect metropolitan productivity? Ind. Innov. 21, 155–177 (2014).

[27] Statistics Sweden, *Construction and Use of Labour Market Areas in Sweden* (Örebro, 2010).

[28] Carlstedt B., Mårdberg B., Construct validity of the Swedish enlistment battery. Scand. J. Psychol. 34, 353–362 (1993). 10.1111/j.1467-9450.2005.00432.x 15660631

[29] Rönnlund M., Carlstedt B., Blomstedt Y., Nilsson L.-G., Weinehall L., Secular trends in cognitive test performance: Swedish conscript data 1970–1993. Intelligence 41, 19–24 (2013).

[30] B. Carlstedt, thesis, University of Gothenburg (2000).

[31] Schich M., Song C., Ahn Y.-Y., Mirsky A., Martino M., Barabási A.-L., Helbing D., A network framework of cultural history. Science 345, 558–562 (2014). 25082701 10.1126/science.1240064

[32] C. R. Shalizi, Scaling and hierarchy in urban economies. arXiv:1102.4101v2 (2011).

[33] L. M. A. Bettencourt, J. Lobo, H. Youn, The hypothesis of urban scaling: Formalization, implications and challenges. arXiv:1301.5919v1 (2013).

[34] J. Mincer, *Schooling, Experience, and Earnings* (Columbia Univ. Press, 1974).

[35] T. Lemieux, in *Jacob Mincer: A Pioneer of Modern Labor Economics*, S. Grossbard, Ed. (Springer, 2006), pp. 127–145.

[36] S. S. Rosenthal, W. Strange, in *Handbook of Regional and Urban Economics, Vol. 4*, V. Henderson, J.-F. Thisse, Eds. (North-Holland, 2004), pp. 2119–2171.

[37] Boschma R. A., Fritsch M., Creative class and regional growth: Empirical evidence from seven European countries. Econ. Geogr. 85, 391–423 (2009).

[38] Statistics Sweden, Swedish standard classification of occupations 1996. Reports on Statistical Co-ordination for the Official Statistics of Sweden (1998).

[39] Yankow J. J., Migration, job change, and wage growth: A new perspective on the pecuniary return to geographic migration. J. Reg. Sci. 43, 486–516 (2003).

[40] Kratz F., Brüderl J., Returns to regional migration: Causal effect or selection on wage growth? Schmollers J. 133, 227–238 (2013).

[41] Romer P. M., Increasing returns and long-run growth. J. Pol. Econ. 94, 1002–1037 (1986).

[42] Bettencourt L. M. A., Samaniego H., Youn H., Professional diversity and the productivity of cities. Sci. Rep. 4, 5393 (2014). 24953448 10.1038/srep05393 PMC4066264

[43] Youn H., Bettencourt L. M. A., Lobo J., Strumsky D., Samaniego H., West G. B., Scaling and universality in urban economic diversification. J. R. Soc. Interface 13, 20150937 (2016). 26790997 10.1098/rsif.2015.0937 PMC4759798

[44] Puga D., The magnitude and causes of agglomeration economies. J. Reg. Sci. 50, 203–219 (2010).

[45] Arzaghi M., Henderson J. V., Networking off Madison Avenue. Rev. Econ. Stud. 75, 1011–1038 (2008).

[46] Larsson J. P., The neighborhood or the region? Reassessing the density-wage relationship using geocoded data. Ann. Reg. Sci. 52, 367–384 (2014).

[47] Bettencourt L. M. A., Lobo J., Strumsky D., Increasing returns to patenting as a scaling function of metropolitan size. Res. Policy 36, 107–120 (2007).

[48] Stumpf M. P. H., Porter M. A., Critical truths about power laws. Science 335, 665–666 (2012). 22323807 10.1126/science.1216142

