# Supplementary material for: Urban scaling and the regional divide
Source: Sci Adv. 2019 Jan 30;5(1):eaav0042. doi: 10.1126/sciadv.aav0042 (PMC6353621; doi:10.1126/sciadv.aav0042)
Supplement: http://advances.sciencemag.org/cgi/content/full/5/1/eaav0042/DC1 [file supp_5_1_eaav0042__index.html]

Science Advances | Science Advances

## Supplementary Materials

**This PDF file includes:**

- Section S1. Full population data, metropolitan areas, and regional composition
- Section S2. Outlier analysis of urban scaling parameters
- Section S3. Replication of the scaling relation’s decomposition with U.S. data
- Section S4. Wage data and measures of individual productivity
- Section S5. Full tabulation of cross-sectional results
- Section S6. Full tabulation of the urban wage premium
- Fig. S1. Scaling relations of urban indicators excluding the three largest labor market areas.
- Fig. S2. Decomposition of the total scaling relation for wages across U.S. Metropolitan Statistical Areas.
- Fig. S3. Complementary analyses of the urban wage premium.
- Table S1. Description of Sweden’s full working-age population.
- Table S2. Creative jobs and the corresponding occupational codes.
- Table S3. Composition effects on the scaling of wage income.
- Table S4. Urban wage premium following a move from one of Sweden’s smaller labor market areas to one of the four largest.

Download PDF

**Files in this Data Supplement:**

- Adobe PDF - aav0042\_SM.pdf
