# Supplementary material for: Urban scaling and the regional divide
Source: Sci Adv. 2019 Jan 30;5(1):eaav0042. doi: 10.1126/sciadv.aav0042 (PMC6353621; doi:10.1126/sciadv.aav0042)
Supplement: http://advances.sciencemag.org/cgi/content/full/5/1/eaav0042/DC1 [file aav0042_SM.pdf]

Science Advances
AAAS

advances.sciencemag.org/cgi/content/full/5/1/eaav0042/DC1

Supplementary Materials for

Urban scaling and the regional divide

Marc Keuschnigg*, Selcan Mutgan, Peter Hedström

*Corresponding author. Email: marc.keuschnigg@liu.se

Published 30 January 2019, *Sci. Adv.* **5**, eaav0042 (2019)
DOI: 10.1126/sciadv.aav0042

This PDF file includes:

Section S1. Full population data, metropolitan areas, and regional composition
Section S2. Outlier analysis of urban scaling parameters
Section S3. Replication of the scaling relation's decomposition with U.S. data
Section S4. Wage data and measures of individual productivity
Section S5. Full tabulation of cross-sectional results
Section S6. Full tabulation of the urban wage premium
Fig. S1. Scaling relations of urban indicators excluding the three largest labor market areas.
Fig. S2. Decomposition of the total scaling relation for wages across U.S. Metropolitan Statistical Areas.
Fig. S3. Complementary analyses of the urban wage premium.
Table S1. Description of Sweden's full working-age population.
Table S2. Creative jobs and the corresponding occupational codes.
Table S3. Composition effects on the scaling of wage income.
Table S4. Urban wage premium following a move from one of Sweden's smaller labor market areas to one of the four largest.

Section S1. Full population data, metropolitan areas, and regional composition

Statistics Sweden, the country's central statistical office, assembled longitudinal geocoded micro-data for us on Sweden's entire population by merging administrative population registers. In Sweden, all register-based information for each individual and each organization gets filed under a unique ID number. The quality of these data is generally very high, with missing data virtually nonexistent.

Table S1. Description of Sweden's full working-age population. (**A**) Differences in population means among Sweden's 71 smaller labor market areas (2,673–234,449 inhabitants), its four largest labor market areas (Stockholm [2.51 million inhabitants], Malmö [1.09], Gothenburg [1.08] and Linköping [0.26]), and its capital Stockholm in 2012. Total population aged 18–60 (in millions); mean age (in years); mean education (in years); mean cognitive ability (as measured in a standardized conscription test [z-std.], for males only); employees in creative jobs (as a fraction of the total labor force). All differences between the smaller and larger labor market areas (LMAs) are significant at $p<0.001$ in two-sided t-tests. (**B**) Stayers (4.77 millions, excluding Stockholm) and those who have left smaller for relatively larger labor market areas (503,274 one-time movers between 1990 and 2012) differ strongly, on average, in education (in years) and cognitive ability (z-std., for males only). Moreover, those with more education tend to self-select into the biggest labor markets, thus passing larger population differences between their native and target labor market areas (pop. diff. in millions). All differences are significant at $p<0.001$ in two-sided t-tests.

**A**	71 smaller LMAs	4 largest LMAs	Stockholm LMA
Population	2.47	2.85	1.47
Age	39.16	38.35	38.34
Education	12.03	12.58	12.67
Cognitive ability	–0.14	0.13	0.19
Creative jobs	26.10	35.55	38.65

**B**	Stayers	Movers
Education	11.16	12.94
Cognitive ability	–0.15	0.26
Pop. diff. | Educ. $\leq$11		0.92
Pop. diff. | Educ. 12–13		1.08
Pop. diff. | Educ. $\geq$14		1.12

Analyses of urban scaling rely on functional rather than administrative boundaries of cities (*3,33*). Similar to the construction of Metropolitan Statistical Areas in the United States, which are frequently used in the urban-scaling literature, Statistics Sweden groups the country's municipalities into unified labor markets based on commuting patterns of local work forces (*27*). These labor market areas cluster around local centers in which <20% of the working population commute to other municipalities and <7.5% commute to any single

neighboring municipality. Each remaining municipality is then assigned to the local center receiving the largest share of its commuters. In 2012, this procedure resulted in the demarcation of 75 highly-modular labor market areas (see `https://tinyurl.com/ycnjj4ew`), which we use as a functional definition for metropolitan areas. Table S1 summarizes composition differences across relevant groupings of labor market areas and highlights movers' contributions to urban labor forces' productivity.

Section S2. Outlier analysis of urban scaling parameters

From the smallest (2,673 inhabitants) to the largest labor market area (2.51 million inhabitants) the scaling relationships reported in Fig. 1 span four orders of magnitude. One could consider the three biggest labor markets, Stockholm (2.51 million inhabitants), Malmö (1.09), and Gothenburg (1.08), outliers in Sweden's urban system. Still, scaling parameters are robust to the exclusion of those three labor market areas (Fig. S1).

Excluding, further, the mining areas Gällivare and Kiruna has little effect on the estimated scaling parameters for company turnover ($\beta = 1.194 \pm 0.054$), property tax ($\beta = 1.170 \pm 0.042$), and the total number of residential moves ($\beta = 1.134 \pm 0.024$), divorces ($\beta = 1.162 \pm 0.062$), college graduates ($\beta = 1.108 \pm 0.024$), and creatives ($\beta = 1.113 \pm 0.018$).

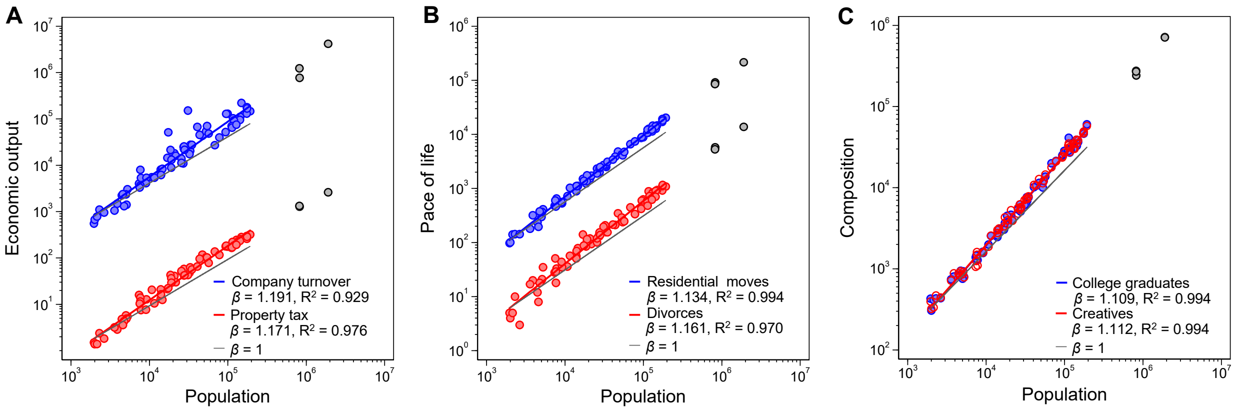

Fig. S1. Scaling relations of urban indicators excluding the three largest labor market areas. (**A**) In this artificially-shrunk urban system, the scaling parameters for company turnover (blue: $\beta = 1.191$ ± 0.056 [95% confidence interval], $R^2 = 0.929$) and collected property tax (red: $\beta = 1.171$ ± 0.042, $R^2 = 0.976$) remain within the range of $\beta \backsimeq 1.15$. (**B**) The same holds for the total numbers of residential moves (blue: $\beta = 1.134$ ± 0.024, $R^2 = 0.994$) and of divorces (red: $\beta = 1.161$ ± 0.062, $R^2 = 0.970$) as well as (**C**) the total numbers of college graduates (blue: $\beta = 1.109$ ± 0.024, $R^2 = 0.994$) and of employees in creative jobs (red: $\beta = 1.112$ ± 0.021, $R^2 = 0.994$). The colored lines show estimates of β from the linearized model (Eq. 1 in Materials and Methods); $M = 72$.

Section S3. Replication of the scaling relation's decomposition with U.S. data

In Fig. S2 we replicate our decomposition of the total scaling relation (Fig. 3) with wage and employment data from the 382 Metropolitan Statistical Areas (MSAs) of the United States. Similar to the Swedish case, the scaling relation for total wages ($\beta = 1.110 \pm 0.022$) is due partly to a higher labor force participation in bigger cities ($\beta = 1.026 \pm 0.011$). Metropolitan Statistical Areas' average wages carry the remaining part of the scaling relation ($\beta = 0.084 \pm 0.014$). Note that further analyses controlling for labor-market productivity related characteristics require full-population micro-data unavailable for the United States.

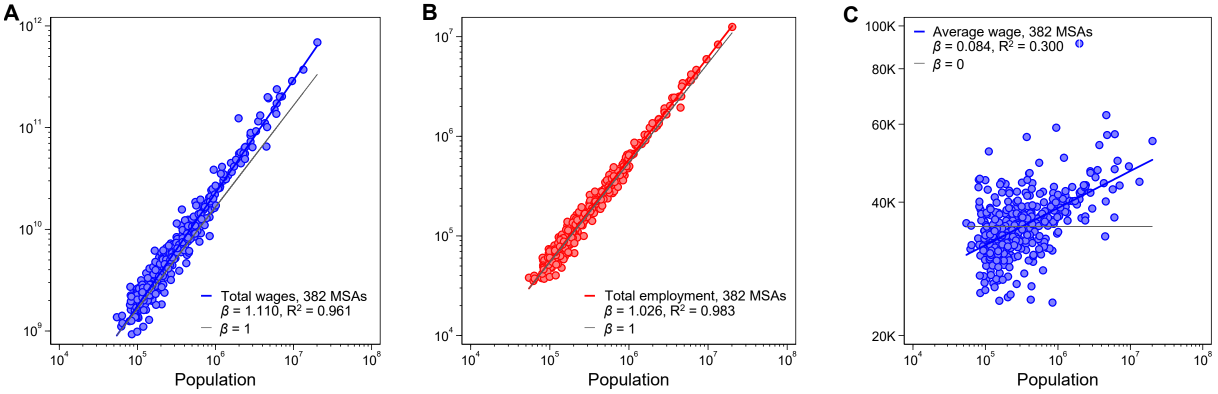

Fig. S2. Decomposition of the total scaling relation for wages across U.S. Metropolitan Statistical Areas. (**A**) The total scaling relation for wages, measured in thousands of US$ in 2015, amounts to $\beta = 1.110$ ± 0.022, $R^2 = 0.961$. (**B**) Also, the total number of employees scales superlinearly with $\beta = 1.026$ ± 0.011, $R^2 = 0.983$. Both colored lines show estimates of β from the linearized model (Eq. 1 in Materials and Methods). (**C**) With $\beta = 0.084$ ± 0.014, $R^2 = 0.300$, per-capita wage carries the remaining part of the total scaling relation ($\beta = 1.026 + 0.084 = 1.110$). Here, we fit Eq. 2 and the gray line indicates a proportional per-capita relation ($\beta = 0$). Data for 2015 obtained from U.S. Bureau of Economic Analysis (`www.bea.gov`).

Section S4. Wage data and measures of individual productivity

Our main analyses (Figs. 3–5) focus on wage earnings as a local source of income. To isolate composition effects, we restrict our complete population data to fully-employed Swedish-born males aged 18–60 who took a compulsory conscription test during 1970–2006 (see below). Instead of giving a description of the entire Swedish population, we thus give priority to clean estimates of the scaling parameter unaffected by, for example, variations in female labor force participation or ethnic discrimination between urban areas of different sizes (see *24* for similar sample restrictions). In our individual-level analyses we also exclude employees in Gällivare and Kiruna (mining areas in the far north), as their economies depend almost exclusively on the extraction of natural resources. In our cross-sectional (2012) and longitudinal analyses (1993–2012) we scrutinize the same set of 1.29 million individuals nested in 73 labor market areas.

The strong norm of equality that prevails in Sweden places bounds on income differences. This is most likely the explanation for why $\beta = 1.082 < 1.15$ for Swedish wages (Fig. 3A). Payment regulations cap the dispersion of wages between regions and may weaken the superlinearity of total wages across Sweden's labor market areas. Recall that our aim lies not in demonstrating $\beta \simeq 1.15$ for Swedish wages but in exploring how much of β is independent from direct composition effects and thus consistent with the interconnectivity explanation. Although we look at a relatively small economy, the interconnectivity explanation is a general theory that should also hold for Sweden.

In our cross-sectional analysis we partial out composition effects on the scaling of wages, first by considering the human capital earnings function (*34,35*), which models individual log(wage) as the sum of years of education and a quadratic function of years of work experience. Both education (12.5 years on average, min=9, max=20, sd=2.3) and work experience (17.1 years on average, min=0, max=22, sd=6.0) are directly observable in the register data. Second, unlike seminal net-agglomeration studies from regional economics (*24,25*), our data permit inclusion of a measure of cognitive ability and thus crucially extend the vector of observed individual characteristics. Third, as an additional feature of urban composition we include a binary variable measuring each employee's innovativeness. In the following, we describe these measures in detail.

We draw on standardized test results of cognitive ability among male conscripts. The Swedish military enlistment procedure consisted of a series of physical, psychological, and intellectual tests all men had to take at age 18–19 until its abolition in 2010. The enlistment procedure included an assessment of cognitive ability similar to the Armed Forces Qualifying Test (AFQT) used in the United States (*28*). There were separate paper and pencil tests for verbal understanding, technical comprehension, spatial ability, and logic. Each test consisted of 40 items presented in order of increasing difficulty and speed. With only minor revisions implemented over the years, this procedure evaluated the same four underlying abilities throughout the entire period (*29*). Motivation for participation in military service was explicitly not a factor for evaluation and avoiding enlistment by obtaining low-ability scores was not possible. Our data combine the test results with a normally distributed scale ranging from 1–9, which has been found to be a good measure of general intelligence (*30*). Enlistment became less comprehensive after 2006 and the numbers dropped substantially. We thus restrict our analyses to men who went through the enlistment procedure between 1970 and 2006, which, for the year 2012, leaves us with a 81.6% coverage of Swedish men in the relevant age bracket 18–60. To account for test revisions over time, we z-standardize each year's scores. This results in a bell-shaped distribution of cognitive ability for the years 1970–2006 with mean 0 and standard deviation 1.

Table S2. Creative jobs and the corresponding occupational codes. We capture creative employees in the sense of Florida (*18*), relying on the International Classification of Occupations (ISCO-88) codes provided in (*37*) which permit an almost 1:1 mapping to the Swedish Standard Classification of Occupations (SSYK96) available in our data (*38*).

**Creative core**	SSYK96
Physicists, chemists, and related professionals	211
Mathematicians, statisticians, and related professionals	212
Computing professionals	213
Architects, engineers, and related professionals	214
Life science professionals	221
Health professionals (except nursing)	222
College, university, higher education teaching professionals	231
Secondary education teaching professionals	232
Primary and preprimary education teaching professionals	233
Special-education teaching professionals	234
Other teaching professionals	235
Archivists, librarians, and related information professionals	243
Social sciences and related professionals	224 + 249
Public service administrative professionals	247
**Creative professionals**	
Legislators, senior officials, and managers	1
Nursing and midwifery professionals	223
Business professionals	241 + 248
Legal professionals	242
Physical and engineering science associate professionals	31
Life science and health associate professionals	32 + 5135
Finance and sales associate professionals	341
Business services agents and trade brokers	342
Administrative associate professionals	343
Social work associate professionals	346
**Bohemians**	
Writers, musicians, creative or performing artists	245
Photographers, image/sound recording equipment operators	3131
Artistic, entertainment, sports associate professionals, journalists	347
Fashion and other models	521

In line with our composition argument, Florida (*18*) stresses the role of highly-skilled creative workers for urban productivity. Following his classification, we define creative employees as “people in science and engineering, architecture and design, education, arts, music, and entertainment whose economic function is to create new ideas, new technology, and new creative content” as well as those with occupations in “business and finance, law, health care, and related fields [...] engaging in complex problem solving” (p. 8). We capture these individuals utilizing the International Classification of Occupations (ISCO-88) codes provided in (*37*) which permit an almost 1:1 mapping to the Swedish Standard Classification of Occupations (SSYK96; *38*) available in our data (Table S2). We assign the value 1 to

each employee in a creative occupational category (0 for employees in all other occupations). In our restricted data, 47.1% work in creative jobs. The number of creatives scales with $\beta = 1.112 \pm 0.017$ across Sweden's labor market areas (see Fig. 1C; note that Fig. 1 refers to the full population of Sweden).

Alternately, one may interpret the number of individuals in creative jobs as an approximation of a city's relative position in an urban system of occupational location (*21,32*). Correspondingly, the share of creatives in the full working-age population is larger in Stockholm (38.7%) than in the four largest labor markets combined (35.6%) and substantially larger than in the rest of the country (26.1%; Table S1A). Florida's concept thus resonates with the "theory of central places" (*21*) according to which specialist industries with high economic returns locate in central places where they can distribute fixed costs over larger catchment areas.

Section S5. Full tabulation of cross-sectional results

To arrive at a net-agglomeration effect for Swedish wages (Fig. 3C), we estimate individual-level log(wage) regressions and include our proxies for individual productivity (see Eq. 3 in Materials and Methods). Table S3 summarizes our results.

Table S3. Composition effects on the scaling of wage income. Two-level random-effects regressions (Eq. 3 in Materials and Methods) with 1.29 million individuals (level 1) nested in 73 labor market areas (level 2) in 2012. N = labor market population. Cluster-robust standard errors in parentheses. Individuals working in the mining areas Gällivare and Kiruna are excluded. All estimates are significant at p<0.001.

	(1) Human capital		(2) Cognitive ability		(3) Creative job		(4) Central industries		(5) Population density	
log(N)	.0382	(.004)	.0364	(.004)	.0283	(.005)	.0346	(.004)		
×high density									.1046	(.011)
×low density									.0271	(.005)
Education	.0765	(.004)	.0644	(.003)	.0353	(.003)	.0615	(.002)	.0336	(.002)
Experience	.0340	(.003)	.0365	(.003)	.0322	(.002)	.0364	(.002)	.0322	(.002)
Experience^2	−.0004	(.000)	−.0005	(.000)	−.0005	(.000)	−.0005	(.000)	−.0005	(.000)
Ability			.0549	(.005)	.0238	(.002)	.0489	(.003)	.0228	(.002)
Creative job					.3320	(.023)			.3241	(.016)
Central industry							.1222	(.033)		
R^2	.086		.091		.127		.095		.130	

The control variables carry the expected weights, with education, experience, ability, and creative job characteristics correlating positively with log(wage). Further, in line with the expectation of the human capital earnings function, the negative sign of the quadratic

term of experience implies a bound on the returns to professional experience. All estimates are significant at p<0.001. The step-wise inclusion of the controls increases the explained variance of log(wage) from 8.6 (model 1) to 12.7% (model 3), indicating that each control adds to the prediction of individual wage notwithstanding their collinearity: The linear correlation between education and cognitive ability is $\rho = 0.496$ and each variable correlates with the binary indicator of creative job characteristics at $\rho = 0.481$ and $\rho = 0.396$, respectively (p<0.001 for all pairwise correlations). Most importantly, the inclusion of observable worker characteristics associated with individual productivity reduces the elasticity between wage and city size to 0.0283 (model 3). This net-agglomeration effect is well in line with studies from regional economics controlling for characteristics of local industries and workforces (*24,25,36*) and matches—based on a continuous indicator—the per-capita increase to size ($\beta = 0.028$, yet insignificant) reported for U.S. patenting activity (*47*).

Additional model specifications substantiate our results: In model 4, we demonstrate that substituting our measure of creative job characteristics with a binary variable for central industries—1 for employees in finance, consulting, computation and telecommunication, and media (we follow the classification in [*32*])—adds little to the prediction of log(wage) (+0.4% variance explained compared to +3.6% for the creatives dummy). When further controlling for a creative job title (not tabulated) employment in a central industry shows no significant correlation with log(wage). Hence, the scale effects of central industries appear of lesser importance than individual-level measures of occupational characteristics. Model 5, finally, demonstrates it is not population size *per se* but population density that drives agglomeration effects. Elasticities are much stronger for employees in dense ($\beta = 0.1046 \pm 0.0207$) than in sparse ($\beta = 0.0271 \pm 0.0010$) environments. This finding suggests that density has important effects on individual productivity and corroborates an important assumption of urban-scaling research. This result is based on analyses in which we constructed square catchment areas 500 meters on a side (0.25km^2) surrounding each individual's workplace and counted all other employees therein. We then split the count at the 75% percentile (4,031 employees) and assigned the focal employee to either a low-density (≤4,031 proximate employees) or a high-density environment (>4,031 proximate employees). We sized these catchment areas based on the prior finding that networking effects attenuate quickly and that inadequately disaggregated data may underestimate location benefits (*45,46*). We follow a similar modeling approach ($\log(N) \times$factorial dummy) to estimate urban scaling's social gradients (Fig. 5), splitting the study population in three groups consisting of those with relatively little (<25th percentile), intermediate (25–75th percentile), or high (>75th percentile) education or ability, respectively.

Section S6. Full tabulation of the urban wage premium

Our estimation of the urban wage premium rests on the wage trajectories of individuals who—between 1993 and 2012—left their native labor market areas to work in relatively larger labor markets. We scrutinize the same set of 1.29 million individuals as in the cross-sectional analysis but restrict the estimation of the urban wage premium to individuals who moved only once and had been fully-employed for at least one year both prior and following migration. On average, we observe individual wage trajectories for 14 years, but our unbalanced panel data include individuals with shorter and longer employment histories.

Table S4. Urban wage premium following a move from one of Sweden's smaller labor market areas to one of the four largest. Movers' relative wage change against the counterfactual wage they would have received had they stayed in their native labor markets (Eq. 4 in Materials and Methods). Cluster-robust standard errors in parentheses. Individuals working in the mining areas Gällivare and Kiruna are excluded. All estimates are significant at $p<0.001$.

	(1) Stockholm (2.51 million)		(2) Malmö (1.09 million)		(3) Gothenburg (1.08 million)		(4) Linköping (0.26 million)	
–6 years	.0109	(.009)	.0118	(.015)	.0022	(.010)	−.0133	(.020)
–5 years	.0211	(.010)	.0227	(.016)	.0084	(.011)	.0178	(.023)
–4 years	.0216	(.010)	.0310	(.016)	.0067	(.012)	−.0111	(.025)
–3 years	.0069	(.010)	.0079	(.017)	−.0004	(.012)	−.0082	(.024)
–2 years	−.0184	(.011)	−.0005	(.017)	−.0363	(.012)	−.0384	(.024)
–1 year	−.0714	(.011)	−.0725	(.018)	−.0978	(.013)	−.1055	(.025)
Move	.1676	(.011)	.0437	(.018)	.0650	(.012)	.0428	(.024)
+1 year	.2981	(.011)	.1549	(.017)	.1428	(.012)	.1160	(.024)
+2 years	.3119	(.011)	.1628	(.018)	.1630	(.012)	.1047	(.025)
+3 years	.3366	(.011)	.1720	(.018)	.1608	(.012)	.1271	(.025)
+4 years	.3466	(.011)	.1719	(.018)	.1671	(.012)	.1239	(.025)
+5 years	.3491	(.011)	.1910	(.018)	.1706	(.013)	.1266	(.026)
+6 years	.3605	(.011)	.1978	(.018)	.1701	(.013)	.1184	(.027)
+7 years	.3622	(.011)	.2065	(.018)	.1680	(.013)	.1154	(.028)
+8 years	.3607	(.011)	.2201	(.019)	.1641	(.013)	.1309	(.027)
+9 years	.3658	(.011)	.2075	(.019)	.1585	(.013)	.1176	(.029)
+10 years	.3715	(.012)	.2261	(.020)	.1582	(.014)	.1134	(.028)
Education	.2549	(.002)	.2569	(.002)	.2571	(.002)	.2579	(.002)
Experience	.0662	(.000)	.0660	(.000)	.0659	(.000)	.0658	(.000)
Experience^2	−.0019	(.000)	−.0019	(.000)	−.0019	(.000)	−.0019	(.000)
Fully-employed	.6886	(.002)	.6796	(.002)	.6818	(.002)	.6774	(.002)
Regional GDP	.0022	(.000)	.0022	(.000)	.0022	(.000)	.0022	(.000)
R^2 within	.347		.339		.342		.339	

Table S4 summarizes the results of our within-person distributed fixed-effects regressions. We display the "distributed effects" (*39,40*) on log(wage) following a move from one of Sweden's smaller labor markets to one of the four largest (see Eq. 4 in Materials and Methods).

Both the immediate ($\gamma_{t=1}$) and the long-term urban wage premium ($\gamma_{t=10}$) relate positively to the target area's population size and are most pronounced for those settling into Stockholm's labor market (+29.8% ±2.1 at $t = 1$ and +37.2% ±2.3 at $t = 10$). The control variables show the expected average effects: An additional year of education increases wages by approximately 26%, each year of work experience raises wages by almost 7% (the effect plateaus, as indicated by the negative sign of experience^2), individuals earn around 32% less in years they are not fully-employed, and GDP growth translates into higher wages. Interestingly, many movers experience a slight drop in relative wages shortly before migration, a common finding in the literature (*24*) which may be among the reasons movers choose to leave.

To approximate the interconnectivity effect on individual wages, we are interested in the urban wage premium conditional on population differences between native and target labor market areas. We focus on the long-term urban wage premium ($\gamma_{t=10}$), which includes post-migration earning paths, capturing not only immediate wage benefits of big-city employment but also the accumulation of learning effects in high-density urban environments over time (*24*). In Sweden, moving from any labor market area to a relatively larger area results in $(72 \times 73)/2 = 2,628$ possible combinations of origin and target labor market areas (again, excluding the two mining areas). For the scaling analysis displayed in Fig. 4B—connecting $\gamma_{t=10}$ to log(population difference)—we estimated Eq. 4 (see Materials and Methods) for the 100 labor-market combinations with ≥200 movers in 1993–2012. This yields, for each combination, a separate mean urban wage premium based on respective movers' wage trajectories.

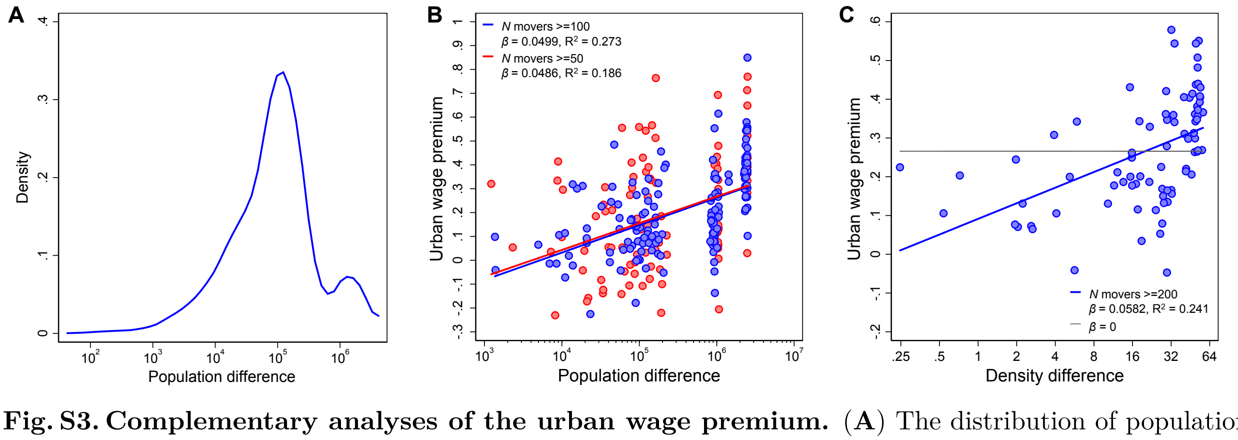

Fig. S3. Complementary analyses of the urban wage premium. (**A**) The distribution of population differences between movers' native and target labor market areas. The hump to the right indicates moves from small places into the three largest labor markets Stockholm, Malmö, and Gothenburg. (**B**) Reducing the necessary number of movers between labor-market combinations leads to parameter estimates very similar to that reported in Fig. 4B: For 165 combinations (blue dots) with at least 100 movers $\beta = 0.050 \pm 0.013$, $R^2 = 0.273$ (blue line); for 260 combinations (blue and red dots) with at least 50 movers $\beta = 0.049 \pm 0.012$, $R^2 = 0.186$ (red line). (**C**) Replacing differences in population size with differences in population density reveals stronger superlinearity as compared to Fig. 4B. The urban wage premium now scales at $\beta = 0.058 \pm 0.023$, $R^2 = 0.241$.

To demonstrate the robustness of the reported scaling parameter ($\beta = 0.050 \pm 0.014$), we perform a similar analysis for origin-and-target combinations sharing at least 50 or 100 movers in 1993–2012 (Fig. S3). The estimates of β are equal to 0.049 $\pm$0.012 and 0.050 $\pm$0.013, respectively. Fig. S3C, again, demonstrates that social density rather than population size drives agglomeration effects: Mean urban wage premiums plotted against the difference in population density between native and target labor market areas (measured as the difference in the number of inhabitants per km^2; mean=5.7, min=–40, max=58) reveals stronger superlinearity ($\beta = 0.058 \pm 0.023$) as compared to the baseline in Fig. 4B.
